# Ileal obstruction caused by migration of deflated intragastric balloon after bariatric surgery treated with laparotomy and semicircular ileotomy: Case report

**DOI:** 10.1016/j.ijscr.2024.109997

**Published:** 2024-07-03

**Authors:** Adeodatus Yuda Handaya, Polycarpus David Subroto, Azriel Farrel Kresna Aditya

**Affiliations:** a Digestive Surgery Division, Department of Surgery, Faculty of Medicine, Universitas Gadjah Mada/Dr. Sardjito Hospital, Yogyakarta 55281, Indonesia; b Faculty of Medicine, Universitas Gadjah Mada/Dr. Sardjito Hospital, Yogyakarta 55281, Indonesia

**Keywords:** Bariatric surgery, Semicircular ileotomy, Intragastric balloon, Small bowel

## Abstract

**Introduction:**

Obesity is a major global health issue with serious consequences, including death. The intragastric balloon (IGB) is a bariatric surgery option but is limited to 6–12 months due to risks such as deflation, migration, and, rarely, intestinal obstruction. These complications are difficult to diagnose and manage.

**Presentation of case:**

A 46-year-old woman with an intragastric balloon for ten months experienced gastric distension, excessive salivation, and nausea, leading to hospitalization. Abdominal radiography and a CT scan revealed a small bowel obstruction caused by the balloon, located 40 cm before the ileocecal junction. A laparotomy was performed to explore the surgical site further. An ileotomy was conducted to remove the balloon during the surgery. The patient was discharged in stable condition after five days.

**Discussion:**

Complete small bowel obstruction due to intragastric balloon migration in bariatric surgery is very rare. Initial symptoms include mid-gut dilation, nausea, and vomiting. A CT scan is the best method to locate and identify the cause of intragastric balloon migration. Laparoscopy can be challenging in acute obstruction cases due to limited space, increasing the risk of iatrogenic bowel injury. Therefore, laparotomy with a semi-circular ileotomy is a safe treatment option.

**Conclusion:**

Ileal obstruction due to intragastric balloon migration is a rare but serious complication of bariatric surgery, which requiring immediate surgical intervention. The use of a semi-circular ileotomy during laparotomy has proven to be an effective and safe treatment option for complete obstruction.

## Highlights

• Intragastric balloons (IGB) are used in bariatric therapy but limited due to risks like deflation, migration, and intestinal blockage.
• Laparotomy with semicircular ileotomy is a safe surgical option for complete small bowel obstruction caused by IGB migration.
• Prompt and appropriate surgical intervention is essential to prevent life-threatening complications from ileal obstruction due to IGB.

## 1 Introduction

Obesity is recognized as a global issue that poses significant health risks and contributes to high mortality rates. There are many weight loss strategies available, but none are risk-free. The intragastric balloon (IGB), a well-known temporary weight loss therapy, has emerged as a promising option for some patient populations [1,2]. Its use as a bridging therapy in bariatric surgery, particularly for those with severe obesity, is said to reduce perioperative problems and technical challenges while shortening surgery time [3].

Guidelines recommend a treatment duration of 6–12 months for IGB, depending on the type of balloon, due to the increased risk of deflation and subsequent migration. While mild side effects such as nausea, vomiting, and abdominal pain are common, serious complications include gastric ulcers, small bowel obstruction, gastric perforation, and, in rare cases, death [4]. Patients with small bowel obstruction typically present to the emergency department with cramping and colicky abdominal discomfort, often accompanied by nausea, vomiting, and constipation [5,6]. This report describes a case where a patient presented with signs and symptoms of gastric obstruction due to a deflated and migrated intragastric balloon. This study has been reported according to SCARE 2023 criteria [7].

## 2 Case report

**Fig. 1:**
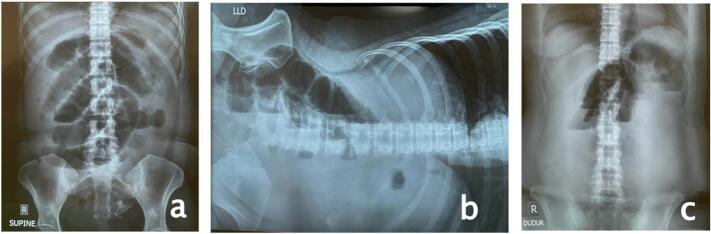
Central dialataion of small intestine on abdominal radiograph a. AP Supine b LLD c. semi erect shows small Bowel obstruction.

**Fig. 2:**
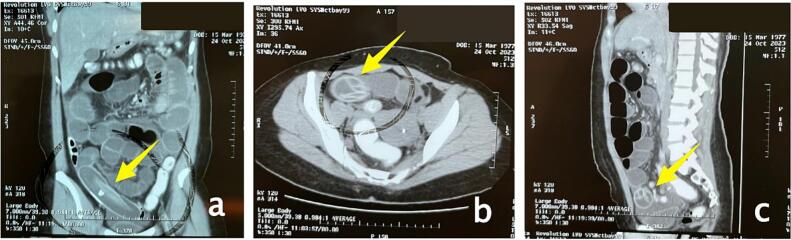
MSCT abdomen showed corpal deflated ballon intraluminal of small bowel caused small bowel obstruction in right lower quadran showed in a. Coronal b axial c and sagital view.

**Fig. 3:**
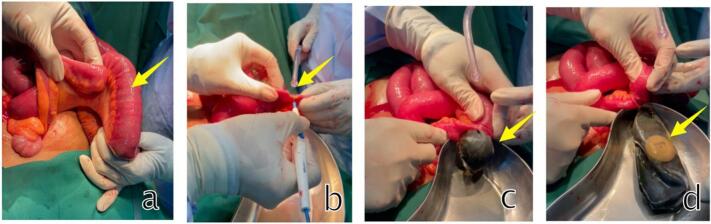
Intraoperative Finding a. Intralunimal defalted ballon b. semicircular incision c. evacuated ballon d. after ballon evacuated.

A 46-year-old female patient arrived at the emergency department with abdominal distension and excessive salivation but no fever. Three days before admission, the patient experienced nausea and an upset stomach. Her family doctor diagnosed her with gastritis and treated her, but her symptoms did not improve. Upon admission to the emergency department, abdominal imaging showed small bowel obstruction (Fig. 1) with an unknown cause, and there were no signs of surgery on the abdominal wall. The patient had used an intragastric balloon for ten months, inserted at another hospital. After one day in the ward, a CT scan (Fig. 2) revealed small bowel distension and the presence of a gastric balloon. During exploratory laparotomy, the surgical team found the balloon in the small bowel system, 40 cm proximal to the ileocecal junction. A semi-circular ileotomy was performed to evacuate the balloon (Fig. 3), followed by repair with 2/0 Vicryl sutures. After receiving food and antibiotics for five days, the patient was discharged in stable condition. Follow-up examinations in the first and second weeks post-operation showed the patient's condition was satisfactory, the surgical wound was healing well, and no complaints were reported.

## 3 Discussion

Obesity is a global epidemic that causes numerous diseases and leads to high mortality rates. Many weight loss procedures exist; however, none are entirely risk-free. The intragastric balloon (IGB) has been used for decades as a temporary weight loss intervention and can be a valuable strategy for certain individuals [1]. The insertion of an IGB typically results in an average total weight loss of about 7–17 kg. The total weight loss (TWL) percentage ranges from 7 to 15 %, and the excess weight loss (EWL) percentage ranges from 22 to 50 % when the balloon is removed. One year after removal, patients usually maintain a weight loss of about 7–9 kg, equating to 8–9 % TWL and 29–33 % EWL. Additionally, IGB placement has been shown to improve metabolic syndrome, including type 2 diabetes mellitus, hypertension, hyperglycemia, and fatty liver disease. Consistent results were observed in a study with a one-year follow-up. However, the effectiveness of IGB is significantly lower compared to other commonly performed laparoscopic bariatric procedures, such as adjustable gastric banding (AGB), sleeve gastrectomy, and Roux-en-Y gastric bypass [3]. The use of IGB as a temporary treatment in bariatric surgery, especially for patients with extreme obesity, is expected to reduce perioperative complications, process complexity, and surgery duration [3].

Patients often choose the intragastric balloon (IGB) procedure to address obesity due to its temporary nature and the perception, advised by doctors, that it carries lower risks compared to other bariatric procedures. In this case, the patient had an IGB in place for ten months before experiencing deflation and migration of the balloon. During this period, no complaints were reported. An endoscopic evaluation six months after insertion showed that the IGB was in excellent condition, and the patient was scheduled for balloon removal after twelve months. Depending on the type of balloon used, the treatment duration for IGB should not exceed six to twelve months due to the increased risk of deflation and migration beyond this period. The most common side effects are nausea and vomiting, with an incidence of 23.3 %, followed by abdominal pain at 19.9 % and gastroesophageal reflux disease (GERD) at 14.4 %. More serious complications include ileal obstruction (0.8 %), gastric ulceration (0.3 %), gastric perforation (0.1 %), and death (0.05 %) [4,5].

Intestinal obstruction is a very rare complication associated with the use of intragastric balloons, with only a few case reports documented in the medical literature [1]. In a study conducted by Ntyl Sondous et al., six patients were examined after having the balloon in place for more than a year before presenting to the emergency department. The longest documented case in this study was four years. In Lopez-Nava's study involving 714 patients, the overall complication rate was 4.1 %. In contrast, Genco's study involving 2515 patients reported an overall complication rate of 2.8 % [6]. Across a total of 4240 patients, obstruction due to deflated balloon migration was found in only 7 cases (0.17 %) [8]. An additional research by Almuhanna A et al. indicated that patients who had an IGB in place for a year and eight months without follow-up might experience balloon deflation and migration [9]. Further investigation revealed that the balloon had deflated and migrated to the ileocolic region, necessitating laparoscopic surgery to address the resulting subacute intestinal obstruction. According to an article by Herve et al., the optimal duration for IGB placement is six months; to reduce the risk of complications, it is recommended to remove the balloon after this period [9].

Early symptoms of a ruptured or deflated balloon typically include blue-colored urine due to the absorption of methylene blue injected into the balloon at the time of insertion [8]. The classic presentation of a patient with small bowel obstruction in the emergency department includes cramping and colicky abdominal pain, often accompanied by nausea, vomiting, and constipation. Seven patients reported abdominal pain without vomiting. The duration of symptoms before seeking medical help ranged from a few hours to seven days [6]. Diagnosis is mostly made in emergency settings, where abdominal X-rays are commonly used to confirm the diagnosis, but CT scans are also useful, especially when complications arise [6]. Computed tomography of the small intestine shows folded structures with a metallic head visible in the loops of the small intestine on the right side of the abdomen [10]. When the diagnosis is uncertain, endoscopy and abdominal ultrasonography are reasonable steps to take [6].

In this case, the patient did not experience blue discoloration of the urine or abdominal colic but reported stomach discomfort, bloating, nausea, and vomiting, which worsened over time. Gastritis did not improve with treatment, leading to the recommendation for surgery. A day after the CT scan, the results showed an image of a foreign body, believed to be a deflated intragastric balloon that had moved into the small intestine. Guidelines state that if intragastric balloon migration is suspected, the patient should undergo a CT scan. This scan will reveal distension in the small intestine and the image of the balloon trapped in the abdominal cavity. Once the diagnosis is confirmed, conservative treatment may be considered if the patient is stable and the deflated balloon can pass through the intestine without obstruction. However, if the patient is unstable or the balloon fails to pass through the intestine within 48 h, surgical intervention is required to remove the balloon. Our patient was evaluated with a CT scan 24 h after the diagnosis, but since her clinical condition did not improve, we decided to perform a laparotomy.

Surgical techniques, whether laparoscopic or open laparotomy, aim to remove the foreign object without causing further damage to the intestine [1]. In this case, we opted for a laparotomy over a laparoscopic approach primarily due to the risk of small bowel injury caused by significant intestinal dilation. In similar cases, it has been necessary to switch from laparoscopy to open mini-laparotomy due to potential peritoneal contamination when making an enterotomy during laparoscopy. This decision was made to minimize the risk of more serious complications [11].

In our case, we performed an ileotomy using a semi-circular incision and repaired it with simple sutures using 2/0 Vicryl to avoid narrowing the lumen after surgery. The initial management strategy for a migrated intragastric balloon causing small bowel obstruction should be chosen based on the location of impaction, the severity of the obstruction, and the expertise of the radiologist, endoscopist, and/or surgeon involved. Balloons causing obstruction in the duodenum can likely be removed endoscopically, while impaction in the jejunum or ileum can be managed with percutaneous needle aspiration (in some cases), endoscopy (double-balloon enteroscopy), laparoscopy, or surgery [11]. In our perspective, laparotomy with semi-circular ileotomy might be an appropriate therapeutic alternative for managing significant small bowel obstruction and dilation caused by extragastric migration of a bariatric IGB located 40 cm proximally to the ileocecal junction.

## 4 Conclusion

Small bowel obstruction with intestine dilatation caused by ileal migrating balloons used in bariatric surgery requires quick and precise surgical intervention to prevent potentially life-threatening complications. Performing a laparotomy with a semicircular incision has emerged as a safe and effective alternative treatment option.

## Consent

Written informed consent was obtained from the patient for publication of this case report and accompanying images. A copy of the written consent is available for review by the Editor-in Chief of this journal on request.

## Ethical approval

This study obtained approval from the Institutional Review Board of the Faculty of Medicine, Public Health, and Nursery Universitas Gadjah Mada with letter number KE/FK/0315/EC/2024.

## Funding

The authors declare that this study had no funding resource.

## Author contribution

Adeodatus Yuda Handaya conceived the study and approved the final draft. Polycarpus David Subroto, Azriel Farrel Kresna Aditya drafted the manuscript and critically revised the manuscript for important intellectual content. Adeodatus Yuda Handaya, Polycarpus David Subroto, and Azriel Farrel Kresna Aditya facilitated all project-related tasks.

## Guarantor

Adeodatus Yuda Handaya.

## Conflict of interest statement

No potential conflict of interest relevant to this article was reported.
